# Mutations in LRRK2 potentiate age-related impairment of autophagic flux

**DOI:** 10.1186/s13024-015-0022-y

**Published:** 2015-07-11

**Authors:** Shamol Saha, Peter E. A. Ash, Vivek Gowda, Liqun Liu, Orian Shirihai, Benjamin Wolozin

**Affiliations:** Departments of Pharmacology, Boston University School of Medicine, Boston, MA 02118 USA; Departments of Medicine, Boston University School of Medicine, Boston, MA 02118 USA; Departments of Neurology, Boston University School of Medicine, 72 East Concord St., Boston, MA 02118 USA

**Keywords:** C. elegans, Autophagy, LRRK2, α-synuclein, Imaging, LC3, Aging

## Abstract

Autophagy is thought to play a pivotal role in the pathophysiology of Parkinson’s disease, but little is known about how genes linked to PD affect autophagy in the context of aging. We generated lines of C. elegans expressing reporters for the autophagosome and lysosome expressed only in dopaminergic neurons, and examined autophagy throughout the lifespan in nematode lines expressing LRRK2 and α-synuclein. Dopamine neurons exhibit a progressive loss of autophagic function with aging. G2019S LRRK2 inhibited autophagy and accelerated the age-related loss of autophagic function, while WT LRRK2 improved autophagy throughout the life-span. Expressing α-synuclein with G2019S or WT LRRK2 caused age-related synergistic inhibition of autophagy and increase in degeneration of dopaminergic neurons. The presence of α-synuclein particularly accentuated age-related inhibition of autophagy by G2019S LRRK2. This work indicates that LRRK2 exhibits a selective, age-linked deleterious interaction with α-synuclein that promotes neurodegeneration.

## Introduction

The large number of neurodegenerative diseases that are associated with the accumulation of insoluble protein aggregates suggests an important role for dysfunction of proteostasis during aging [1]. The two predominant autophagic processes are cell mediated autophagy and macro-autophagy [2, 3]. Increasing evidence suggests that macroautophagy is the predominant process regulating the elimination of the protein aggregates that accumulate in age-related neurodegenerative diseases [4, 5].

Autophagy proceeds through a process of phagophore initiation, assembly, fusion with the lysosome and degradation [6, 7]. Initiation proceeds through pathways mediated by Ulk proteins and beclin/VPS34. Membrane elongation involves a series of Atg proteins (Atg 5, 7, 10 and 12), which prime phospholipids to interact with Microtubule-associated protein 1A/1B-light chain 3 (LC3), form the autophagic membrane, identify ubiquitinated species and engulf the target [7]. The resulting autophagosome then fuses with the lysosome, leading to degradation of the autolysosomal material, including LC3. The appearance of LC3 labeled vesicles is now routinely used to identify autophagy [3].

Increasing evidence suggests that defects in autophagy contribute to the pathophysiology of Parkinson’s disease. Many of the genes associated with familial Parkinson’s disease are required for autophagy. This includes β-glucocerebroside and ATP13A2 [8]. Parkin and PINK1 are two proteins linked to autosomal recessive Parkinsonism that appear to regulate mitophagy [9–11]. These proteins interact to recruit LC3 to mitochondria in peripheral cells, although the applicability of this pathway to neurons remains unclear. α-Synuclein is the principle protein that accumulates in sporadic Parkinson’s disease. α-Synuclein has been shown to interact with the cell mediated autophagy pathway through a process that is inhibited by mutant A53T α-synuclein [12].

Mutations in LRRK2 are common in familial Parkinson’s disease. LRRK2 exhibits pleiotropic functions, perhaps best shown by recent network studies [13]. Recent evidence raises the possibility that the toxic actions of LRRK2 are mediated by α-synuclein [14]. Studies using cell culture first indicated that mutations in LRRK2 interfere with autophagy, including cell mediated autophagy [15–17]. Knockout studies proved that endogenous LRRK2 is required for proper autophagic function [18–20]. The knockout studies were notable for demonstrating strong deficits in autophagic function in the kidney, but autophagic deficits were observed in the dopaminergic neurons or elsewhere in the brain [18–20]. The limited neuronal effects of LRRK2 knockout might reflect compensation by LRRK1, which is a close homologue of LRRK2 present in all mammals.

C. elegans provides a potentially important system to examine the actions of LRRK2 because they have only one LRRK2, termed lrk-1. They also lack endogenous α-synuclein, which enables study of LRRK2 function with or without α-synuclein. We have now used C. elegans to investigate how LRRK2 and α-synuclein affect macroautophagy, and whether the two proteins interact to modify macroautophagy over the nematode lifespan. We created lines of C. elegans that express mCherry fused lgg-1, the nematode homolog of LC3, in dopaminergic neurons, and followed the expression of the lgg-1 reporter throughout the lifespan. We now report that autophagy begins to decline after egg-laying in adults is accomplished. Expressing human mutant LRRK2 enhances the age-related decline. Introducing α-synuclein into the system promotes autophagy at a young age but interacts in a synergistic manner with both WT and mutant LRRK2 to decrease autophagy and promote dopaminergic death in an age-dependent manner. Thus, the interaction between α-synuclein and LRRK2 interferes with cellular function predominantly in aging tissues.

## Results

### Lgg-1::mCherry reflects autophagic activity in DA neurons

**Fig. 1:**
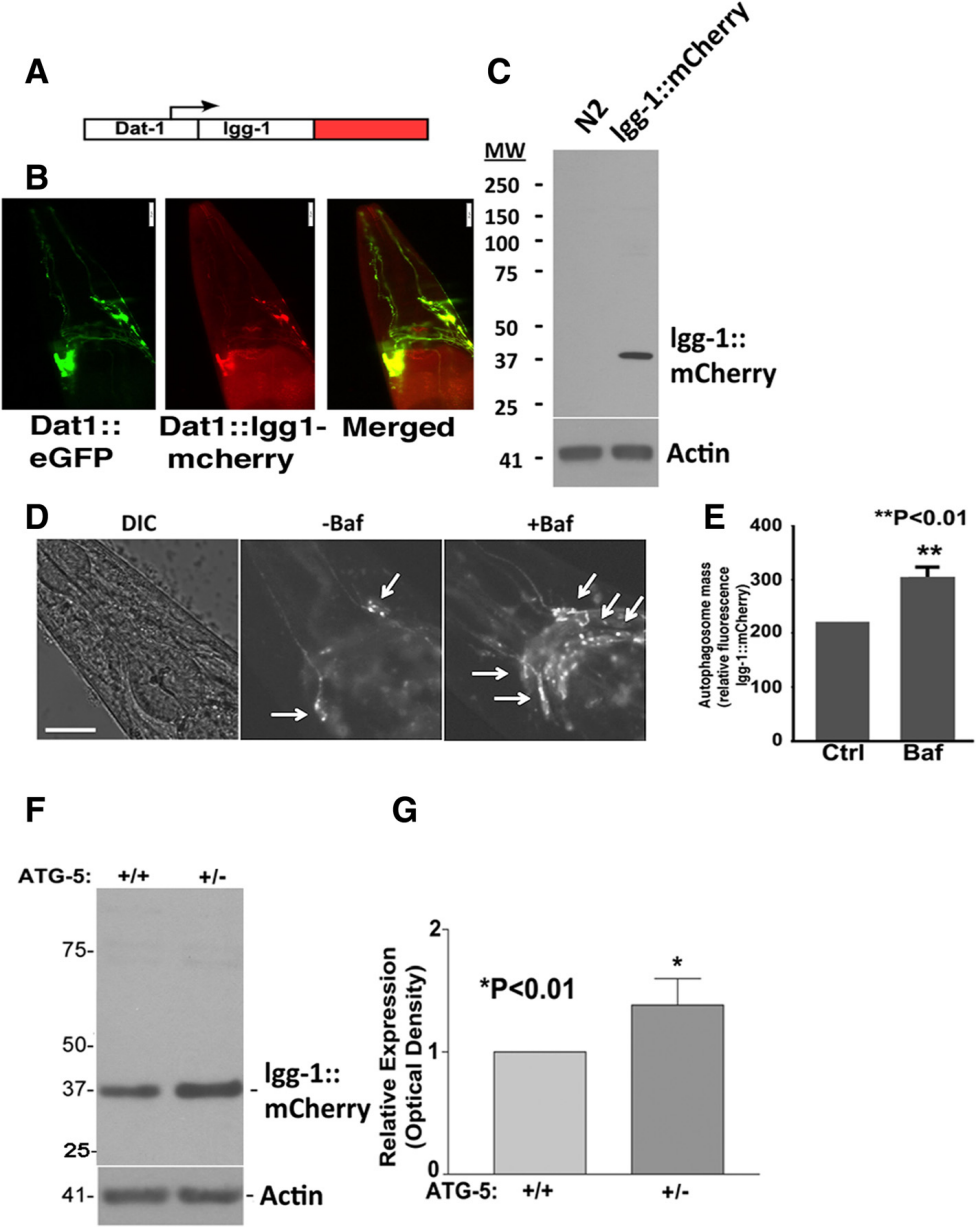
Generation of the *wlz56 dat-* *1*::lgg-1::mCherry C. elegans line. **a**. Structure of *dat-1*::lgg-1::mCherry reporter construct. **b**. Expression of *dat-1*::lgg-1::mCherry construct in dopaminergic neurons. The left panel shows the expression pattern of *dat-1::*GFP, the middle panel shows the expression pattern of *dat-1*::lgg-1::mCherry, and the right panel shows the merged images demonstrating the overlapping expression. **c**. Immunoblot showing expression of lgg-1::mCherry, which shows a monomeric band at approximately 37 kD (arrow). **d**. Levels of the *dat-* *1*::lgg-1::mCherry reporter reflect autophagic flux. Exposure of *wlz56* to bafilomycin (100 μg/ml, 72 h) increases fluorescence. Arrows point to puncta of *dat-* *1*::lgg-1::mCherry fluorescence. **e**. Quantification of the lgg-1::mCherry reporter fluorescence showing increased expression in nematodes treated with 100 μg/ml bafilomycin for 72 h. **f**. Deletion of Atg5 increases lgg-1::mCherry protein. Immunoblot showing total *dat-1::lgg*-1::mCherry protein from 50 crossed heterozygous F1 males. Control contains males (F1) from N2 crossed with *wlz56* (*dat-* *1*::lgg-1::mCherry) while Atg5 contains males (F1) from Atg5 mutant strain crossed with *wlz56.* A monomeric 37KD band represents the *dat-1::lgg*-1::mCherry fusion protein. **g** Total lgg-1::mCherry band intensities were scanned and quantified lines containing WT or mutated ATG5 (n = 8). Scale bar = 20 μm

To create an optical reporter for autophagic activity, we generated a construct consisting of the dopamine transporter (dat-1) promoter driving lgg-1, the C. elegans homolog of LC3, which was fused to mCherry (Fig. 1a). Nematodes carrying the *dat-1*::lgg-1::mCherry arrays were selected, integrated by irradiation and backcrossed 6 times to remove unwanted mutations. Line *wlz56* was selected for further study based on strong expression of lgg-1 observable by imaging and immunoblot (Fig. 1b, c). Upon imaging, the lgg-1::mCherry gave a strong signal in the soma, and appeared as smaller puncta in processes of the dopaminergic neurons of C. elegans (Fig. 1b). By immunoblot the lgg-1::mCherry was apparent as a monomer located at approximately 39 KD (Fig. 1c). The size of the lgg-1::mCherry chimeric protein is consistent with the size expected for the combination of native lgg-1 (12.3 KD) plus mCherry (27 KD). Prior studies indicate that LC3 and lgg-1 can be cleaved by ATG4 *in vitro*, however knockdown studies suggest that this does not occur to a significant degree in cultured neurons or *in vivo* [21, 22]. Immunoblots of the lgg-1::mCherry did not show any significant amount of cleavage fragments (Fig. 1c), which is consistent with the knockdown studies suggesting that there is little lgg-1 cleavage in neurons *in vivo* [21].

Next we examined whether line *wlz56* (*dat-1*::lgg-1::mCherry) line responded appropriately to known autophagic modulators. First we examined the response to bafilomycin, which is an inhibitor of the ATPV6 hydrogen pump [23]. The nematodes expressing *dat-1*::lgg-1::mCherry were bleached and age-synchronized eggs hatched in liquid media containing 100 μg/ml bafilomycin. After 24 h treatment, the nematodes were transferred to NGM plates containing bafilomycin and maintained until images were taken (adult day 2). Maintenance on bafilomycin increased levels of *dat-1*::lgg-1::mCherry, which is consistent with hypothesis that the *dat-1*::lgg-1::mCherry reporter reflects autophagic function (Fig. 1d & e).

To independently test the responsiveness of the *dat-1*::lgg-1::mCherry reporter line to changes in autophagic flux, we crossed the *wlz56* line (carrying the *dat-1*::lgg-1:: mCherry reporter) with a defective ATG-5 (otn8052) line. Atg-5 is required for formation of the autophagosome, and without it, lgg-1 remains dispersed. Crossed heterozygous males (F1) expressing *dat-1*::lgg-1::mCherry and a single copy of mutant ATG-5 exhibited increased levels of lgg-1::mCherry which is consistent with reduced autophagic flux leading to reduced degradation of lgg-1::mCherry (Fig. 1f, g). These data indicate that the reporter line accurately reflects the status of the autophagic system in dopaminergic neurons.

### Autophagy becomes increasingly impaired with aging

**Fig. 2:**
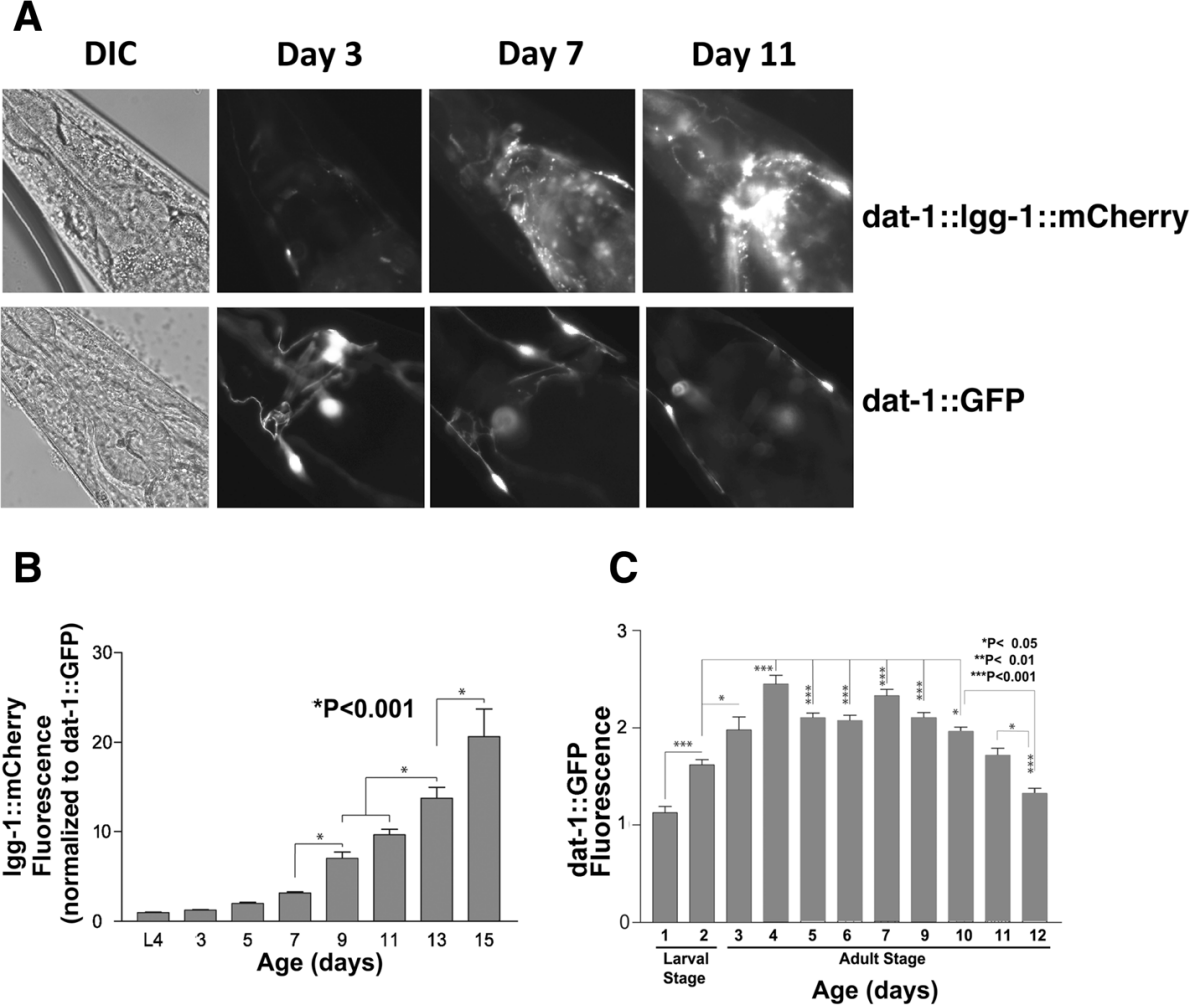
Quantification of autophagic flux over the lifespan. **a**. Representative pictures from the *wlz56 dat-1*::lgg-1::mCherry and *dat-1*::GFP lines at varying ages. **b**. Total mCherry fluorescence from four anterior CEP neurons and the lateral regions of the head ring was captured in age synchronized *dat-1*::lgg-1::mCherry nematodes over much of the nematode lifespan (N = 15, for each time point). The fluorescence was normalized to corresponding data captured from age-synchronized C. elegans carrying *dat-1*::GFP to control for differences in the size of DA neurons as the nematodes matured and aged (Fig. C). **c** *dat-1*::GFP fluorescence over nematode lifespan. Total GFP fluorescence from all four CEP neurons are plotted to quantify the pattern of *dat-1*::GFP expression (N = 15, for each time point). The increased fluorescence at young ages reflects the increasing size of the neurons, while the decreased fluorescence at older ages reflects age-related degeneration of the dopaminergic neurons. The mean fluorescence data were used to correct the age and cell size data for lgg1-mCherry expression for Fig. 3

Having established that the *dat-1*::lgg-1::mCherry reporter reflects autophagic flux, we preceded to examine how autophagy varies over the life cycle. First we crossed the *wlz56* line (*dat-1*::lgg-1::mCherry) with a line expressing GFP driven by the *dat-1* promoter (*dat-1*::GFP, BY200) [24]. Nematodes from the resulting cross were age-synchronized and both mCherry and GFP fluorescence were followed over time. The *dat-1*::lgg-1::mCherry fluorescence (Fig. 2a & b) was normalized to the amount of *dat-1*::GFP fluorescence (Fig. 2c) to control for changes in size and number of the dopaminergic (DA) neurons over the lifespan. Interestingly, *dat-1*::lgg-1::mCherry expression increased with age; the increase did not result from differences in expression from the dat-1 promoter, because fluorescence from the corresponding *dat-1*::GFP reporter increased from days 1–4 (reflecting growth of the nematode), was level between days 4–7, and decreased between days 7–12 (Fig. 2c). Thus, the *dat-1*::lgg-1::mCherry reporter showed a steady increase in expression, even after normalization to DA neuronal size by comparison to the *dat-1::*GFP reporter (Fig. 2a, b & c). This indicates that autophagic flux (*dat-1*::lgg-1::mCherry fluorescence) varies markedly over the life cycle, exhibiting progressive age-related deficits.

### Mutations in LRRK2 Impair Autophagy

Development of the *dat-1*::lgg-1::mCherry reporter line offers the opportunity to investigate how LRRK2 affects autophagy *in vivo*. We previously created lines of nematodes expressing human WT and mutant (G2019S, R1441C and Kinase Dead, KD) LRRK2 driven by the pan-neuronal synaptobrevin-1 promoter (*snb*::LRRK2) [25]. Our prior studies demonstrated that expressing human LRRK2 corrects deficits caused by loss of the endogenous nematode lrk-1 gene, which indicates that human LRRK2 can complement nematode lrk-1 function [25]. To investigate how LRRK2 affects autophagy, we crossed the *wlz56* line with lines carrying LRRK2 (WT, G2019S, R1441C and kinase dead, KD) and a line carrying an allele with a deletion in lrk-1 (km17, referred here after as Δlrk-1). The crossed lines were bred to homozygocity for *snb*::LRRK2(WT, G2019S, R1441C, KD and Δlrk-1) and *dat-1*::lgg-1::mCherry.

**Fig. 3:**
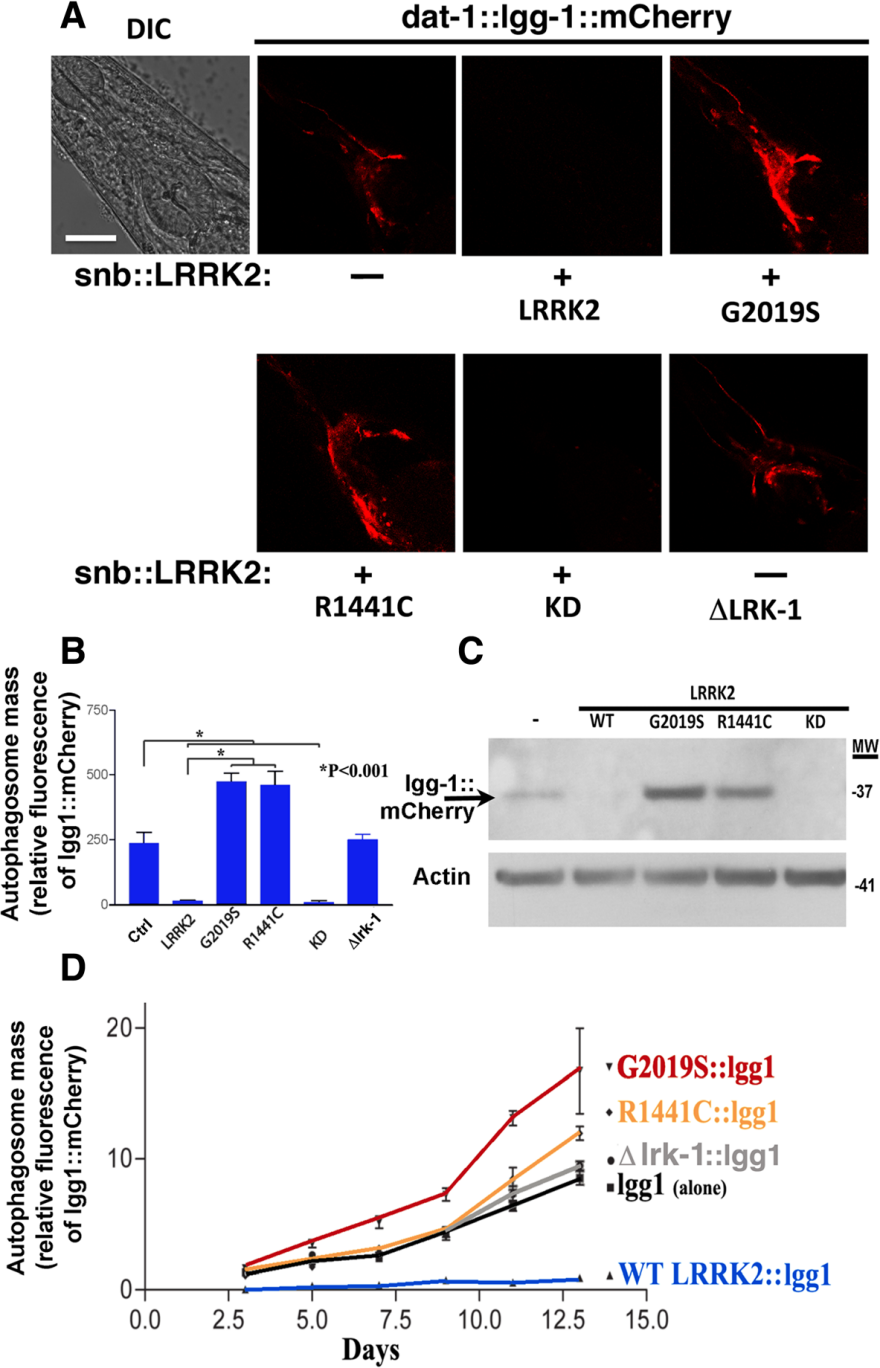
LRRK2 modifies autophagic flux. **a**. Fluorescence of the *dat-1*::lgg-1::mCherry reporter in nematode lines co-expressing *snb-1*::LRRK2. Expressing WT and kinase dead (KD) LRRK2 greatly increases autophagic flux, while G2019S or R1441C expression decreased autophagic flux, causing a corresponding increase in fluorescence. Scale bar = 20 μm. **b**. Quantification of total fluorescence from four CEP neurons and lateral part of nerve ring for each of the nematode lines (Ctrl, lgg-1 and lgg-1/LRRK2 (WT, KD, G2019S and R1441C) at day 5 of life. **c**. Immunoblot showing total lgg1::mCherry protein derivatives from the age synchronized whole animals at day 5 of age. The arrow points to lgg1::mCherry. **d**. Total mCherry fluorescence from four CEP neurons and lateral part of nerve ring captured in age synchronized nematode lines (Ctrl, lgg-1 and lgg-1/LRRK2 (WT, G2019S anR1441C) over much of the lifespan. N = 40 animals/condition, **P < 0.001 and *P < 0.05 compared to lgg-1::LRRK2 (WT). lgg-1::LRRK2 (G2019S) differed from lgg-1 only at day 13 (P < 0.05). lgg-1::LRRK2 (R1441C or Δlrk-1) differed from lgg-1::LRRK2(WT) at time points of 7 days or greater

The *dat-1::*lgg-1::mCherry reporter was used to examine the effects of LRRK2 genotypes on autophagy in age-synchronized lines at day 5 (adult day 3, Fig. 3a & b). Expressing LRRK2 decreased lgg-1::mCherry levels by over 90 %, suggesting improved autophagic function (Fig. 3a & b). In contrast, expressing the *dat-1::*lgg-1::mCherry in the Δlrk-1 line (which lacks the kinase and WD domains) caused no change in *dat-1::*lgg-1::mCherry, producing a level equivalent to the *wlz56* (*dat-1::*lgg-1::mCherry) line, which does not express human LRRK2 (Fig. 3a & b). Expressing WT or KD LRRK2 both reduced *dat-1::*lgg-1::mCherry levels, suggesting increased autophagic flux. Next, we examined the effects of the G2019S and R1441C LRRK2 genes on lgg-1::mCherry levels. Fluorescence of both mutant lines was 90 % higher than with the *dat-1::*lgg-1::mCherry and *dat-1::*lgg-1::mCherry/*km17* (Δlrk-1) lines, and over 100-fold higher than for the *dat-1::*lgg-1::mCherry /*snb*::LRRK2 WT line (Fig. 3a & b). These results suggest that disease-linked mutations in LRRK2 cause a striking impairment of autophagic function.

To confirm that the changes in *dat-1::*lgg-1::mCherry fluorescence reflected the protein levels, we also examined levels of *dat-1::*lgg-1::mCherry in age-synchronized nematode lines at day 4 by immunoblot (Fig. 3c). The results obtained by immunoblot paralleled the imaging results. Analysis of monomeric lgg-1::mCherry levels indicated that the *dat-1::*lgg-1::mCherry/LRRK2 WT and KD lines exhibited greatly reduced levels, while the *dat-1::*lgg-1::mCherry/LRRK2 G2019S and R1441C lines both exhibited increased lgg-1::mCherry levels. In addition, the *dat-1::*lgg-1::mCherry/Δlrk-1 line exhibited levels similar to that of the *wlz56* line. Thus, quantification by imaging and immunoblot both indicate that WT and KD LRRK2 reduce lgg-1::mCherry, while G2019 and R1441C LRRK2 increase lgg-1::mCherry. These results support the hypothesis that LRRK2 modulates autophagic flux in C. elegans.

### Mutant LRRK2 causes progressive deficits in autophagy throughout the lifecycle

Because of the importance of aging in PD, we were curious to understand how disease-linked mutations in LRRK2 might affect autophagy over the life cycle. Each of the nematode lines was synchronized and aged by passage every other day. Expression of the *dat-1::*lgg-1::mCherry reporter was quantified as described above. Levels of *dat-1::*lgg-1::mCherry increased throughout the lifespan for all of the lines (Fig. 3d). The of rank order of *dat-1::*lgg-1::mCherry expression among the lines remained similar throughout the lifespan, with lines carrying G2019S LRRK2 exhibiting the highest levels and those carrying WT LRRK2 exhibiting the lowest levels (Fig. 3d). However, the *dat-1::*lgg-1::mCherry levels in the WT LRRK2 line remained very low throughout the lifespan, while the other lines exhibited high levels by day 10. These data point to strong effects of LRRK2 on autophagy through the lifespan.

### LRRK2 does not affect a lysosomal reporter

**Fig. 4:**
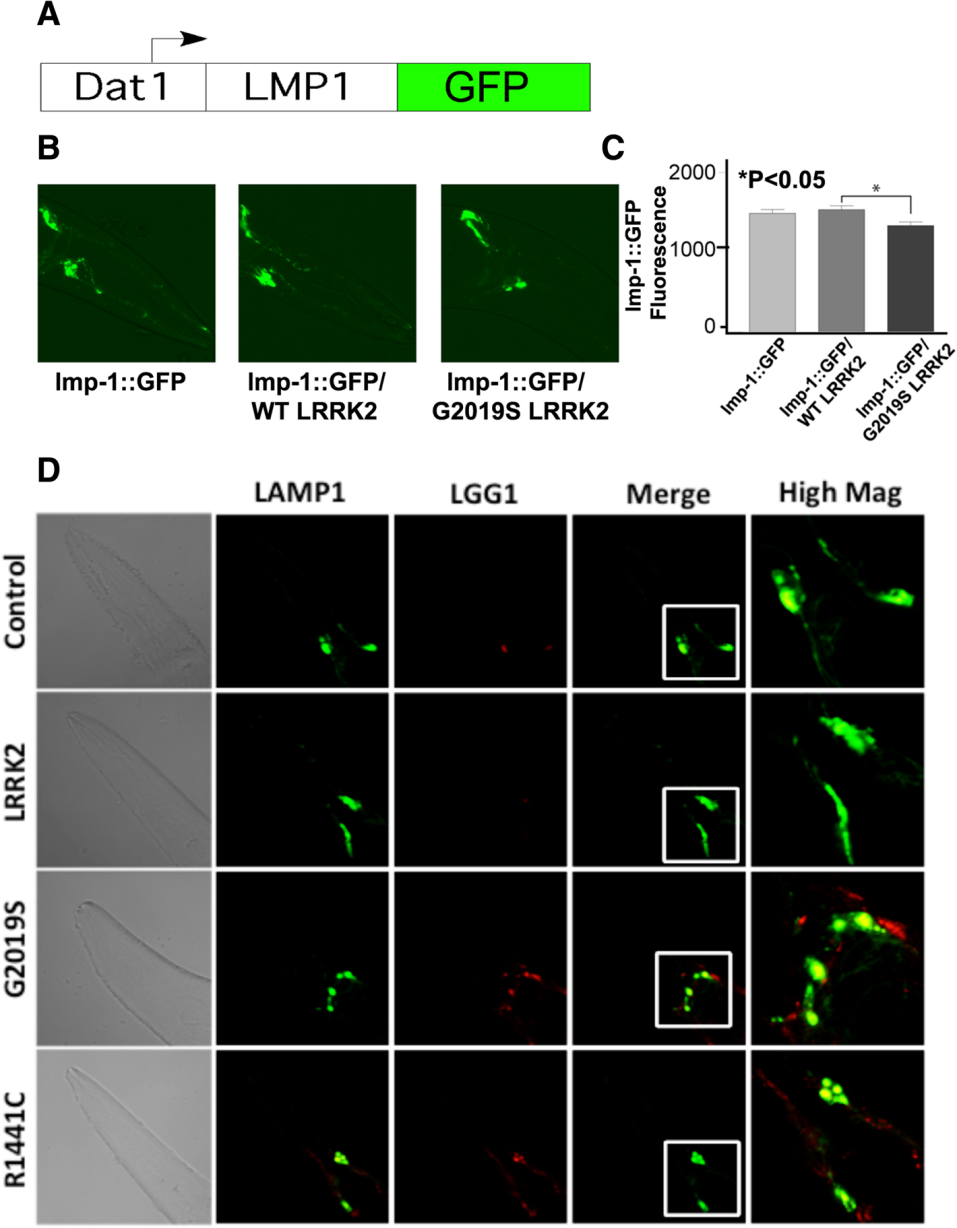
Generation of the *dat-* *1*::lmp-1::GFP C. elegans line. **a**. Structure of *dat-1*:: lmp-1::GFP reporter construct. **b**. Expression of *dat-*1::lmp-1::GFP construct in dopaminergic neurons of day 3 adult nematodes. The lmp-1::GFP identifies locations of lysosomal vesicles in the anterior deirid (ADE) and CEP dopaminergic neurons and in their dendrites. Scale bar = 40 μm. **c**. Quantification of lmp-1::GFP fluorescence of the *dat-* *1*::lmp-1::GFP lines and lines crossed to the *snb-1*::LRRK2 (WT or G2019S) lines. **d**. Co-expressing the *dat-* *1*::lmp-1::GFP construct with *snb*::LRRK2 (WT or G2019S) changes GFP fluorescence only marginally while mCherry fluorescence shows robust changes in response to the type of LRRK2 transgene expressed (adult Day 3). Scale bar = 40 μm. The insets (white boxes) are shown in the adjacent column at higher magnification

To understand whether LRRK2 affects lysosomal function in a manner similar to autophagic function, we generated a reporter for lysosomal function, and crossed that reporter to the LRRK2 lines. We generated a reporter for the C. elegans homologue of the LAMP1 adapter, lmp-1, which is a lysosomal surface protein. The entire lmp-1 coding sequence was fused to eGFP and placed under control of the *dat-1* promoter (Fig. 4a). This line expressed *dat-1*::lmp-1::GFP (labeled *wlz57*), and exhibited strong expression in dopaminergic neurons (Fig. 4b). The *wlz57,* lmp line was crossed to the *snb*::LRRK2 lines (WT and G2019S), and fluorescence was examined at day 3 of adulthood in synchronized nematodes. Co-expressing WT LRRK2 with the lmp-1 reporter did not change the *dat-*1::lmp-1::GFP levels (Fig. 4b & c). Expressing G2019S LRRK2 caused a decrease in the lmp-1::GFP fluorescence, but the decrease was modest (Fig. 4b & c). These data contrasted with the results observed for the lgg-1/LRRK2 lines, where WT and mutant LRRK2 exhibited levels of fluorescence differing by almost 100-fold (Fig. 3). Crossing of the lmp-1 and lgg-1 lines emphasized the differences (Fig. 4d). lmp-1::GFP expression was readily evident in all of the lines, while the lgg-1::mCherry expression was extremely low in the *dat-1::*lgg-1::mCherry/LRRK2 WT line. In addition, imaging the reporters showed that much of the lgg-1::mCherry in the G2019S localized to non-overlapping areas adjacent to the lmp-1::GFP,, suggesting impairment of autolysosomal fusion in this line (Fig. 4d). The contrast between the moderate changes in lmp-1::GFP expression in LRRK2 lines and the dramatic changes in lgg-1::mCherry expression in the LRRK2 lines provides support for a hypothesis that the effects of LRRK2 on lgg-1::mCherry levels reflect selective impairment of the autophagic system.

### Mutant LRRK2 exacerbates age-related deficits in autophagy in C. elegans expressing α-synuclein

**Fig. 5:**
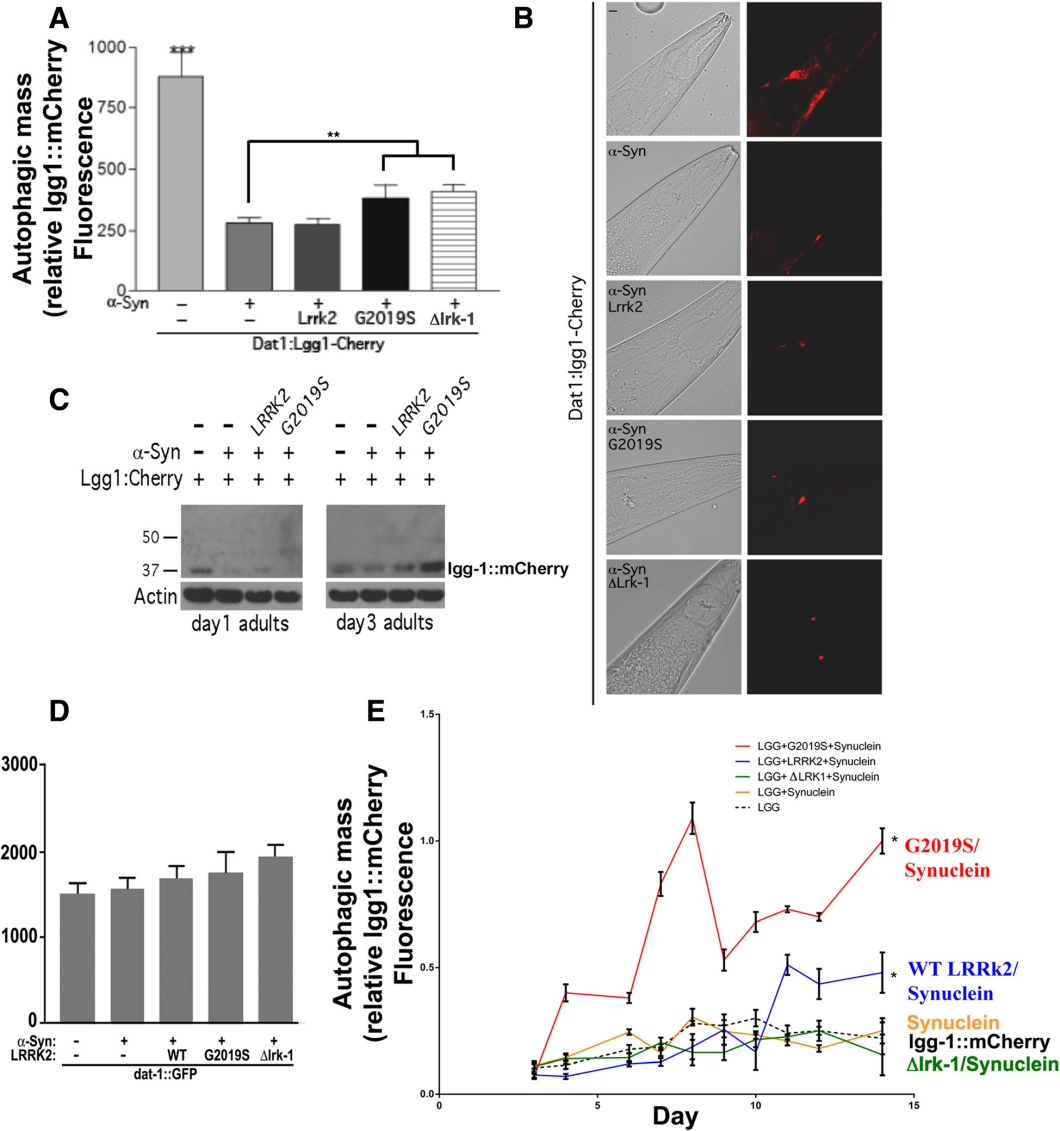
α-Synuclein interacts with LRRK2 to regulate autophagy. **a**. Total lgg-1::mCherry fluorescence in four CEP neurons and nerve ring from 5 day (adult day 3) nematode lines expressing α-synuclein and LRRK2 (WT, G2019S or Δlrk-1). **b**. Representative pictures of *dat-* *1*::lgg-1::mCherry fluorescence in C. elegans lines expressing α-synuclein and LRRK2 (WT, G2019S or Δlrk-1). Scale bar represents 60 μm. **c**. Immunoblot of lgg-1::mCherry in lines of C. elegans lines expressing α-synuclein. Age-synchronized nematodes (50 nematodes/lane, adult days 1 and 3) were used. Actin is shown below to indicate relative protein load per well. **d**. A control experiment showing no change in levels of *dat-1*::GFP fluorescence in C. elegans lines expressing α-synuclein and LRRK2. Total fluorescence from all four CEP neurons from day 3 adult nematodes were plotted to detect any nonspecific general effects from α-synuclein and LRRK2 expression. **e**. Levels of lgg-1::mCherry fluorescence over the lifespan in C. elegans lines expressing α-synuclein ± LRRK2 (WT, G2019S or Δlrk-1). Total fluorescence of *dat-* *1*::lgg-1::mCherry from CEP neurons and nerve ring was measured during a 14 day period using age synchronized nematodes. Total *dat-* *1*::lgg-1::mCherry fluorescence was normalized to *dat-1*::GFP fluorescence in lines expressing α-synuclein ± LRRK2 (WT, G2019S or Δlrk-1), shown in panel D. The scales for the experiments in Figs. 3D and 5E are not meant to be quantitatively identical because the experiments were performed at different times. N = 40 animals/condition, **P < 0.001 and *P < 0.05 compared to lgg-1::mCherry. The G2019S LRRK2/α-synuclein line also differed from the WT LRRK2/α-synuclein line at the 9 and 10-day time points at P < 0.05

The differential effects of LRRK2 on the autophagosome compared to the lysosome highlight the autophagosome as a key organelle that is sensitive to disease-linked mutations in LRRK2. Autophagy is also known to be sensitive to α-synuclein expression, with mutant α-synuclein interfering with cell-mediated autophagy, and aggregated α-synuclein inhibiting macroautophagy [12, 26]. Thus, we were curious to determine how expressing α-synuclein affects the response of nematode autophagy to LRRK2 expression. C. elegans represents a particularly intriguing model to study the effects of α-synuclein because it lacks any endogenous homologue of α-synuclein. We began our studies by creating triple crosses expressing the *dat-1::*lgg-1::mCherry reporter, α-synuclein (*dat-1*::syn) and LRRK2 (WT or G2019S). The synuclein line used was that previously reported by the Caldwell laboratory [27], while the LRRK2 lines were those described above and reported previously by us [25].

lgg-1::mCherry levels were quantified optically and by immunoblot (Fig. 5a, b & c). Co-expressing α-synuclein (*dat-1*::syn) with *dat-1*::lgg-1::mCherry caused a striking reduction in the amount of mCherry levels observed by fluorescence and immunoblot (Fig. 5a, b & c). The decrease could not be explained simply by promoter competition, because expressing *dat-1*::syn with *dat-1*::GFP did not change levels of GFP (Fig. 5d). The selective reduction of *dat-1*::lgg-1::mCherry associated with α-synuclein expression indicates that expressing α-synuclein increases autophagy in young adult (day 1) nematodes.

Next we examined whether the presence of α-synuclein modified the *dat-1*::lgg-1::mCherry levels in nematodes expressing both α-synuclein and LRRK2 in adult nematodes (day 3). Since the LRRK2 lines are driven by the synaptobrevin promoter, addition of the *dat-1*::syn transgene would not be expected to alter LRRK2 transcription. Expressing α-synuclein WT LRRK2 had no effect on lgg-1::mCherry levels, while G2019S/R1441C LRRK2 caused only modest increases in lgg-1::mCherry levels (Fig. 5a, b & c). These data suggest that α-synuclein improves autophagic flux along the same pathway as LRRK2.

We proceeded to examine how aging affected the expression of the *dat-1*::lgg-1::mCherry reporter line in the presence of α-synuclein and LRRK2 (WT, G2019S, R1441C and Δlrk-1). The results demonstrated a striking interaction between α-synuclein and LRRK2. After adult day 5 the line expressing G2019S LRRK2 (*dat-1*::lgg-1::mCherry/*dat-1*::syn/*snb*::G2019S LRRK2) began exhibiting increased *dat-1*::lgg-1::mCherry expression (Fig. 5e). The line expressing WT LRRK2 also exhibited increased *dat-1*::lgg-1::mCherry levels with aging, however the increase occurred at a later age (beginning about day 10) and was smaller in size (Fig. 5e). In contrast, levels of the *dat-1*::lgg-1::mCherry, the *dat-1*::lgg-1::mCherry/*dat-1*::syn and the *dat-1*::lgg-1::mCherry/*dat-1*::GFP/Δlrk-1 lines did not show as strong age-dependent increases (Fig. 5e). These data suggest that in the presence of α-synuclein both WT and G2019S LRRK2 promote an age-related dysfunction of autophagy. For WT LRRK2, this age-related deficit in autophagy is elicited by the presence of α-synuclein, while the G2019S LRRK2 mutation enhances the age-related dysfunction in the presence or absence of α-synuclein.

### LRRK2 enhances age-dependent α-synuclein toxicity in C. elegans

**Fig. 6:**
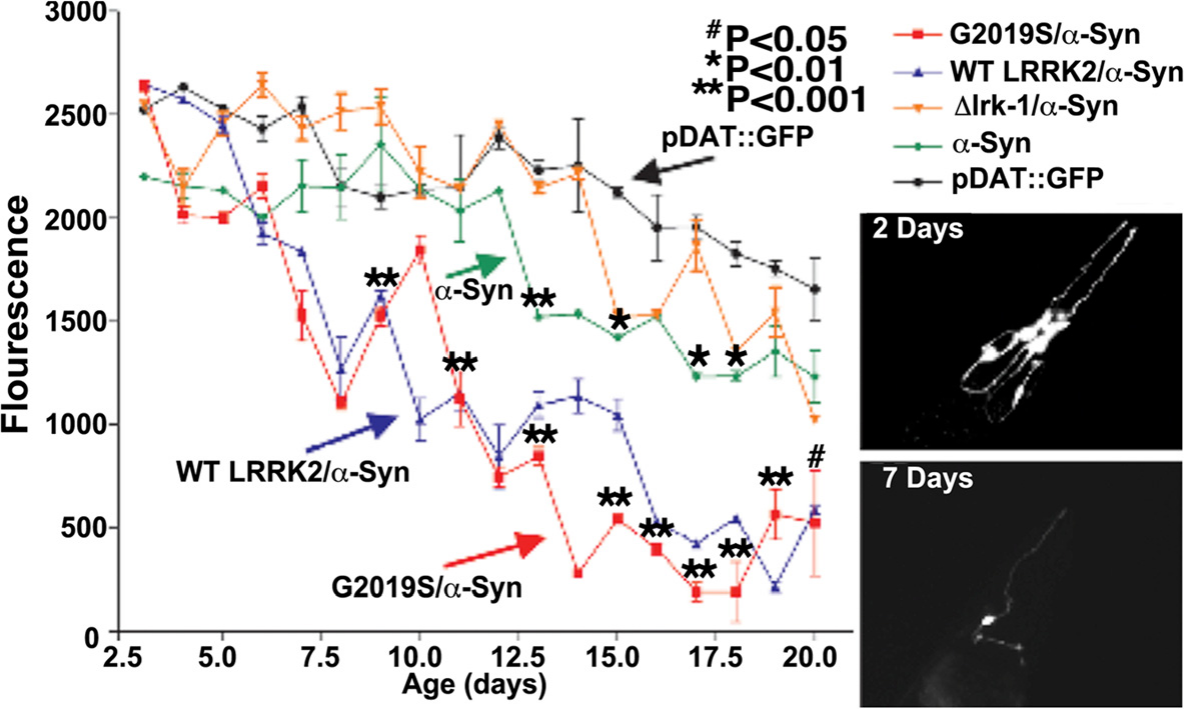
Survival of dopaminergic neurons in C. elegans lines expressing α-synuclein and LRRK2. The expression profile of a *dat-1*::GFP reporter was followed to monitor the fluorescence intensity of CEP neurons over much of the lifespan in C. elegans lines expressing α-synuclein ± LRRK2 (WT, G2019S or Δlrk-1). Inset: Photos from representative adult day 2 and adult day 7 nematodes expressing *dat-1*::GFP/*dat-1*::α-Synuclein/*snb::*G2019S LRRK2 showing the fluorescence of GFP in the CEP neurons and nerve ring. N = 30 animals/condition, **P < 0.001, *P < 0.01 and ^#^P < 0.05 compared to *dat-1*::GFP. The G2019S and WT LRRK2/α-synuclein lines also differed from the α-synuclein line at days 16–20 at P < 0.001

α-Synuclein is thought to contribute to degeneration of dopamine neurons. Thus, we tested whether nematodes expressing α-synuclein ± LRRK2 might exhibit impaired dopamine neuronal survival over the lifespan. We investigated dopamine neuron survival in lines expressing LRRK2 (WT, and G2019S), α-synuclein and *dat-1*::GFP (Fig. 6). Dopaminergic neurons expressing WT α-synuclein and LRRK2 (WT or G2019S) exhibited extensive degeneration, with almost a complete loss of dopaminergic neurons by 17 days of age (Fig. 6). The amount of degeneration associated with expressing G2019S LRRK2 (and WT α-synuclein) appeared to be greater than that for WT LRRK2 (plus α-synuclein), but the difference did not reach the level of statistical significance (Fig. 6). Dopaminergic neurons expressing only WT α-synuclein also showed a modest but statistically significant increase in degeneration of dopaminergic neurons (25 ± 5 %, Fig. 6), but the amount of degeneration was much less than that observed with LRRK2 and α-synuclein co-expression. Thus, LRRK2 potentiates degeneration of dopaminergic neurons associated with α-synuclein in dopaminergic neurons in C. elegans.

## Discussion

The current study presents a new tool for studying autophagy in C. elegans, and then uses this tool to evaluate the interactions between LRRK2, α-synuclein, autophagy and aging in dopaminergic neurons. The generation of LC3::mCherry provides a valuable reporter for monitoring autophagic flux. The LC3::mCherry reporter has been used extensively in mammalian systems, and is widely accepted as an accurate reporter of autophagic flux [28]. In extending the reporter to the nematode, we used lgg-1, which is the nematode homolog of LC3, to insure that it would interact appropriately with the nematode autophagic system. The lgg-1 construct was designed using the dopamine transporter promoter, which drives selective expression in dopaminergic neurons. Restricting expression to the eight DA neurons simplifies the complexity of the visual field, and allows analysis of autophagy in the specific neuronal type that is most affected by the pathophysiology of PD.

A large number of studies indicate that levels of LC3 are inversely proportional to autophagic flux [3, 7]. The current study used an lgg-1 (nematode LC3 homolog) construct driven by the *dat-1* promoter. We quantified lgg-1 levels by fluorescence intensity (Figs. 1, 2, 3, 4 and 5), immunoblotting (Fig. 1, 3 and 5) and finally by counting puncta. Quantification of the strength of *dat-1* promoter activity over the lifespan showed a modest effect of the aging process. Fluorescence from the *dat-1*::GFP promoter decreased with aging, which was opposite to the increase in fluorescence observed with the *dat-1*::lgg-1::mCherry reporter. This indicates that increases in the lgg-1 reporter with aging did not reflect age-related increases in activity of the *dat-1 *promoter. The increase in activity of the lgg-1::mCherry reporter with bafilomycin and ATG-5 deletion were also consistent with prior studies in mammalian cells, in which deficits in autophagy increase activity of the LC3::GFP reporter, which suggests that lgg-1::mCherry levels correlate with autophagic flux. The number of lgg-1::mCherry granules also reflects changes in autophagy, increasing with bafilomycin treatment as autophagic flux becomes stalled (Fig. 1a) and reflecting genotype status in nematodes expressing LRRK2 and/or α-synuclein (Fig. 4d). An additional concern was lgg-1 cleavage. One prior study show that lgg-1 can be cleaved at the C-terminus *in vitro,* however neither our study nor a prior study observed evidence of significant cleavage *in vivo* [21, 22]. Work from Alberti et al. shows that lgg-1 and lgg-2 exhibit functional overlap with respect to autophagy and complement the autophagic activity of the companion protein [29]. Finally, we also generated a *dat-1*::lmp-1::GFP lysosomal reporter. The readout from this reporter provides a strong comparison with the *dat-1*::lgg-1::mCherry reporter, and was striking because it exhibited no changes in response to expression of LRRK2 constructs; these results support the hypothesis that the changes in lgg-1::mCherry reporter reflect autophagic flux rather than transcription from the *dat-1* promoter or other factors. Thus, multiple independent lines of evidence support the hypothesis that the lgg-1::mCherry reporter reliably reflects autophagic flux.

We characterized autophagy over the lifespan, and observed progressive age-related inhibition of autophagy once the nematodes had finished their reproductive period. WT LRRK2 increased autophagic flux in young nematodes, while mutant LRRK2 (G2019S and R1441C) inhibited autophagy. We observed that the *dat-1::*lgg-1::mCherry reporter was responsive to concomitant expression of LRRK2 constructs, while introducing α-synuclein into C. elegans dopamine neurons increased autophagy in young adult nematodes, and the effect of α-synuclein was dominant over concomitant expression of LRRK2 (WT or mutant). During aging, both mutant LRRK2 and α-synuclein inhibited autophagy and increased dopaminergic degeneration. Although these proteins are beneficial at young ages, competitive actions of LRRK2 and α-synuclein on similar uptake systems might impede removal of α-synuclein aggregates, producing a synergistic inhibition of autophagy, a corresponding accumulation of insoluble, oligomeric α-synuclein and synergistic increases degeneration of DA neurons. In addition, although WT LRRK2 improves autophagy throughout the lifespan when expressed in absence of α-synuclein, co-expressing α-synuclein with WT LRRK2 lead to an age dependent inhibition of autophagy, and a synergistic increase in degeneration of DA neurons. These data suggest that LRRK2 and α-synuclein affect autophagy through interacting pathways that lead to synergistic effects, and supports other studies suggesting they might act through similar pathways [14].

LRRK2 and α-synuclein are both known to modulate vesicular function [30–36]. Knockout of LRRK2 in the mouse reduces autophagic flux in the mouse kidney [18]. In addition, a LRRK2 regulatory network that we recently developed indicates that several autophagy linked genes are part of the LRRK2 network, including VPS-34 and HDAC-6 [37]. Interpretation of WT LRRK2 over-expression studies in mammalian cells is less clear because the effects are frequently modest. However, WT LRRK2 expression in C. elegans produces a striking reduction in lgg-1::mCherry levels, suggesting increased autophagic flux. The stronger effect of LRRK2 in nematodes might reflect the simpler biology of these organisms. Nematodes (and *drosophila*) have only one LRRK, lrk-1. LRRK2 might possess a stronger ability to impact on the autophagic system than lrk-1, which would mean that expressing LRRK2 in the nematode induces a strong gain of function.

The actions of α-synuclein and LRRK2 that we observed fit well with the prior studies suggesting that α-synuclein and LRRK2 promote vesicular function, and also fits well with our clinical understanding of the pathophysiology of PD. The increase in autophagy that we observed induced by expressing α-synuclein in young adult nematodes is consistent with other studies showing that α-synuclein promotes vesicular dynamics. Loss of α-synuclein inhibits formation of synaptic vesicles, and reduces dopaminergic function [30–32]; conversely, expressing α-synuclein can compensate for the deleterious of CSPα deletion at the synapse [38]. Analysis of α-synuclein actions might be particularly striking in C. elegans because it lacks an endogenous homolog of α-synuclein, thus the transgene is introducing a novel function to the nematode.

LRRK2 also exhibits a biology that is appears linked to vesicular dynamics. LRRK2 is associated with vesicles and endosomal uptake [33, 36]. WT LRRK2 showed a strong ability to increase autophagy throughout the lifespan, which might also reflect activity directed towards vesicular functions, although many pathways regulate autophagy. In contrast, mutant LRRK2 (G2019S and R1441C) inhibits autophagy, which is similar to reports by other groups as well [15, 17, 25, 39–41]. The ability of both G2019S and R1441C LRRK2 to decrease autophagy below that of nematodes lacking even endogenous nematode lrk-1 points to an activity extending beyond a simple loss of function, and suggests active inhibition of autophagy. Competition for similar autolysosomal uptake sites would account for the increase in α-synuclein levels upon co-expression with WT or mutant LRRK2, which provides further support that the two proteins act on mutually interacting pathways. With increasing age, this competition appears to also increase levels of oligomeric α-synuclein, leading to enhanced degeneration of dopaminergic neurons. Thus, LRRK2 and α-synuclein appear to act through intersecting pathways, which would be beneficial at young ages, but deleterious at old ages.

The deleterious mix of LRRK2 and α-synuclein becomes increasingly apparent with aging. When autophagy is examined in C. elegans lines expressing LRRK2 without α-synuclein, lines expressing G2019S LRRK2 exhibit a 75 % increase in lgg-1::mCherry fluorescence by day 12 (indicating low autophagic flux), while lines expressing WT LRRK2 maintains consistently minimal fluorescence (indicating rapid autophagic flux). In contrast, in the presence of α-synuclein, lines expressing G2019S LRRK2 exhibit a 4-fold increase in lgg-1::mCherry fluorescence by day 12, while lines expressing WT LRRK2 show almost a doubling of fluorescence and indicating strongly reduced autophagic flux. G2019S LRRK2 and other genetic factors implicated in PD, might impact on a subtle aspect of the autophagic pathway, rather than interfering with autophagy generally. For instance, β-glucocerebrosidase mutations impact on a pathway that appears to selectively affect the ability of neuron to degrade α-synuclein, while also increasing the tendency of α-synuclein to oligomerize [42]. The ability of aggregated α-synuclein to inhibit autophagy suggests a synergistic mechanism in which LRRK2 inhibits degradation of α-synuclein, which leads to the accumulation of oligomeric/aggregated α-synuclein, which adds to age-related autophagic inhibition associated with LRRK2 expression.

The age-related interaction between α-synuclein and LRRK2 also translates to enhanced neurodegeneration. Our prior study showed discordant effects of WT and G2019S LRRK2 on age-linked degeneration of dopaminergic neurons, with WT LRRK2 being protective and G2019S LRRK2 being detrimental [25]. Introducing α-synuclein into the system produces a response that reflects the human condition much better. In the presence of α-synuclein, both WT and G2019S LRRK2 enhance age-linked degeneration of DA neurons. These results show that α-synuclein interacts with both WT and G2019S LRRK2 to cause a synergistic inhibition of autophagy, and an age-linked degeneration of DA neurons. The age-linked degeneration associated with G2019S LRRK2 expression parallels work in mouse, where induced expression of G2019S LRRK2 elicited age-linked degeneration of DA neurons [43]. However, to the best of our knowledge, our report is the first report showing age-linked degeneration of DA neurons associated with expression of WT LRRK2.

## Conclusions

Our study presents a model for the actions of LRRK2 and α-synuclein on autophagy that integrates much of the prior work and provides novel insights into potential age-related interactions of WT LRRK2 and α-synuclein. The ability of WT LRRK2 to enhance degeneration associated with α-synuclein expression provides one of the first examples pointing to a potential deleterious interaction between WT LRRK2 and α-synuclein, such as might occur in sporadic PD. This work also provides a mechanism to test the efficacy of novel therapeutic agents, such as inhibitors of the LRRK2 kinase or α-synuclein aggregation, in the context of aging.

### Methods

#### Plasmid

*dat-1*::lgg-1::mCherry is a full length genomic fusion of lgg-1(Lc3) to mCherry. The stop codon has been removed to attach mCherry as a carboxyl part of lgg-1 fusion protein. The *dat-1*::lmp-1::GPP was also constructed with similar fashion with C. elegans genomic lmp1 gene being fused to a GFP reporter gene. Plasmids were DNA sequenced to make sure that the genes are mutation free and fusions were made as intended. Both mCherry and eGFP constructs were purchased from Addgene.

#### *C. elegans* strains

Transgenic nematodes with autophagic and lysosomal reporters were created by injecting a cocktail of DNAs containing 50 ng/μl of plasmid *dat-1*::lgg-1:mCherry along with wildtype lin-15 plasmid (20 ng/μl) and single stranded DNA (20 ng/μl) to young adults of lin-15 strain, grown at 15 °C. Injected nematodes were grown at 20 °C and muv-less progenies, complemented with wildtype lin-15 gene were selected for further characterization. Stable chromosomally integrated lines were created by γ radiation and backcrossed with Bristol N2 worms (six times) in accordance to standard *C. elegans* protocol.

The other *C. elegans* lines were generated and characterized by our laboratory as described previously [25]. The line expressing wildtype α-synuclein was generously provided by Guy Caldwell (University of Alabama) [44]. The atg-5 (otn8052) deletion line was obtained from the CGC (U. Minn.).

*C. elegans* strains were grown at 20 °C unless other growing temperatures were indicated. Hermaphroditic nematodes were used unless otherwise stated. Nematodes were synchronized either by bleaching method or by letting nematodes laying eggs for three hours. A thin layer of feeding bacteria OP50 was spread on NGM plates or other special plates for all experiments unless otherwise indicated.

Bafilomycin treatment of nematodes was conducted according to Pivtoraiko et al., [23]. 5 % methanol was present in the nematode liquid media for 24 h as part of the bafilomycin treatment.

#### Immunoblotting

Immunoblot analysis was performed with age synchronized nematodes using 15 to 50 nematodes per mini protein gel well. Nematodes were collected in 20 μL of DDH_2_O and combined with equal volume of sample buffer containing 100 mM Na-Tricine pH 7.8, 100 mM DTT, 14 % W/V Glycerol, 4 % lithium dodecyl sulfate (LDS), 0.05 % CHAPS and 0.002 % Bromophenol Blue. Lysis was accomplished by crushing the nematodes with glass pestle in the presence of a small amount of clean sand (one third volume per total nematode volume). The lysates were filtered through columns containing glass beads (425–600 μm) to get rid of cellular debris. The sample buffer containing 4 % LDS (described as above) was added to the lysates in equal proportions and the samples were loaded onto 4-12 % bis glycine SDS page gels (Life Technologies) after incubating them at room temperature for twenty minutes. Protein transfer and probing with specific antibodies were performed according to conventional protocols.

#### Imaging

Images for presentation were taken using a Zeiss LSM710 confocal microscope using an oil immersion 63x αplan-APOCHROMAT objective; Z-stacks covering the depth of the nematode were compressed to yield one image showing the comprehensive expression pattern for lgg-1::mCherry and lmp-1::GFP.

#### Image quantification

Images for quantification were obtained with 40X resolution and saved as Axiovision’s ZVI file format; each data point represents a mean of images from 30 – 50 nematodes. The optical density in each image measured by using Axiovision’s quantitation software program. For quantification of *dat-1*::lgg-1::mCherry, a rectangular box covering the entire nerve area containing the soma of the 4 cephalic (CEP) neurons was generated at each age and used to capture the fluorescence; the same size box was also used to capture corresponding fluorescence of CEP neurons carrying *dat-1*::GFP at each age, as well as background fluorescence of CEP neurons from Bristol N2 nematodes (both red and green channels). The mean background fluorescence was subtracted from the mCherry and GFP fluorescence, and the resulting mean fluorescence from obtained from the *dat-1*::lgg-1::mCherry was then divided by the mean fluorescence from obtained from the *dat-1*::GFP fluorescence to obtain a normalized fluorescence value.

#### Statistical analysis

All results are presented as mean ± SEM with time, treatment and genotype as independent factors. The samples were then analyzed as one or two way ANOVAs, depending on the number of independent variables. When ANOVA showed significant differences, pairwise comparisons between were tested by Newman-Keuls post-hoc testing. Statistical analyses were performed with GraphPad Prism software.

## Abbreviations

DA: Dopamine
Dat: Dopamine transporter
GFP: Green fluorescent protein
lmp-1: LAMP1 adapter
LRRK2: Leucine Rich Repeat Kinase 2
PD: Parkinson disease
Snb: Synaptobrevin
Syn: Synuclein
WT: Wild type
